# (*R*)-Baclofen [(*R*)-4-amino-3-(4-chloro­phen­yl)butanoic acid]

**DOI:** 10.1107/S2056989021012809

**Published:** 2022-01-01

**Authors:** Feodor Belov, Alexander Villinger, Jan von Langermann

**Affiliations:** a University of Rostock, Institute of Chemistry, Biocatalytic synthesis group, Albert-Einstein-Str. 3A, 18059 Rostock, Germany; b University of Rostock, Institute of Chemistry, X-ray structure analytics, Albert-Einstein-Str. 3A, 18059 Rostock, Germany

**Keywords:** crystal structure, (*R*)-baclofen, enanti­opure, hydrogen bonds

## Abstract

This article provides the first single-crystal XRD-based structure of enanti­opure (*R*)-baclofen (form C), C_10_H_12_ClNO_2_, without any co-crystallized substances. In the enanti­opure title compound, the mol­ecules arrange themselves in an ortho­rhom­bic crystal structure (space group *P*2_1_2_1_2_1_). In the crystal, strong hydrogen bonds and C—H⋯Cl bonds inter­connect the zwitterionic mol­ecules.

## Abstract 2

The first single-crystal XRD-based structure of enanti­opure (*R*)-baclofen, a commonly used spasmolytic drug, without any co-crystallized mol­ecules is reported in this article.

## Chemical context

(*R*)-Baclofen, an unnatural β-amino acid and artificial GABA receptor agonist, is a frequently used non-addictive drug to treat muscle spasticity (Dario & Tomei, 2004 ▸). Although baclofen is conventionally applied as a racemic mixture, only the (*R*)-enanti­omer actually mediates a therapeutic effect (Olpe *et al.*, 1978 ▸). In addition, baclofen has been recently approved in France as an alternative medication to treat alcohol dependence (Reade, 2021 ▸). Considering those new developments, the establishment of synthetic routes towards enanti­opure (*R*)-baclofen were discussed recently (Córdova-Villanueva *et al.*, 2018 ▸; Gendron *et al.*, 2019 ▸). 
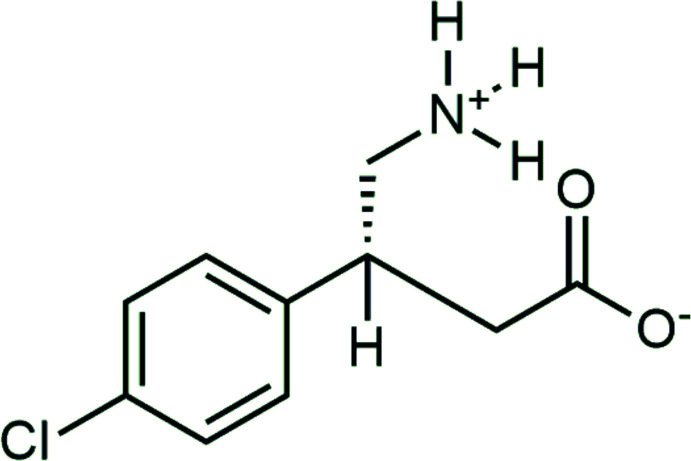


## Structural commentary

**Figure 1:**
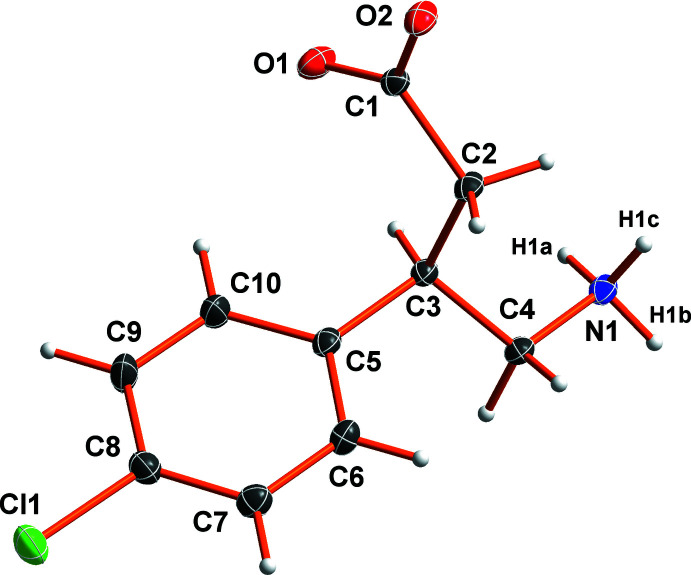
The mol­ecular structure of (*R*)-baclofen with displacement ellipsoids shown at the 50% probability level.

**Figure 2:**
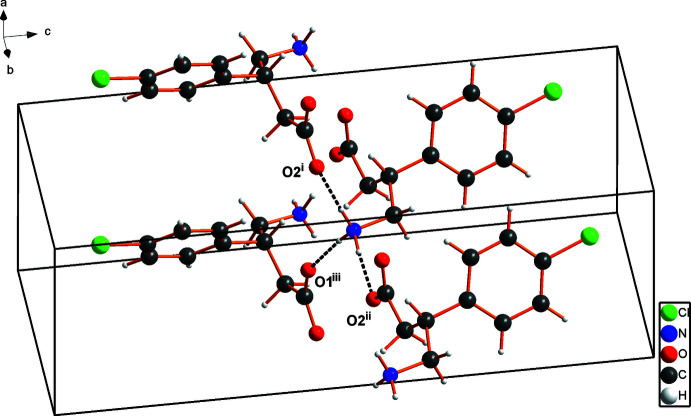
Partial packing diagram of (*R*)-baclofen form *C*.

The mol­ecular structure of the title compound is shown in Fig. 1 ▸. A partial packing diagram is shown in Fig. 2 ▸.

A prediction of crystal forms of the title compound was previously presented by Couvrat *et al.* (2021 ▸), which is based on detailed XRPD-studies and Rietveld refinement. Based on the available XRPD-data, three forms, *A*, *B* and *C*, were observed, of which form *C* is considered to be the most stable form at higher temperatures. The (*R*)-baclofen crystal analyzed in this work corresponds to the newly predicted polymorphic form *C* presented by Couvrat *et al.* (2021 ▸).

The mol­ecules crystallize in a zwitterionic configuration, forming an ammonium and a carboxyl­ate residue. The N-bound hydrogen atoms were located and refined freely. Bond lengths and angles fall into the typically observed ranges for organic mol­ecules without any strain.

## Supra­molecular features

In the crystal of enanti­opure (*R*)-baclofen form *C*, short N—H⋯O hydrogen bonds occur between the carboxyl­ate and the ammonium group of the neighboring baclofen mol­ecule. In parallel, additional hydrogen bonding occurs with neighboring baclofen mol­ecules, resulting in a two-dimensional network parallel to (001), which yields a layered formation of baclofen mol­ecules. Parallel to the hydrogen bonding, T-shaped C—H⋯π inter­actions occur along the layers of aromatic rings within the mol­ecules [C9—H9 ⋯ *Cg*1^viii^= 2.74 Å; C6—H6 ⋯ *Cg*1^ix^= 3.24 Å; symmetry codes: (viii) −*x* + 2, *y* − 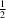 , −*z* + 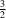 ; (ix) −*x* + 1, *y* + 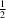 , −*z* + 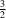 ; *Cg*1 is the centroid of the C5–C10 benzene ring] . The inter­action planes both form angles of 67° with the plane of the corresponding benzene ring (C5–C10).

The combination of both effects yields the observed structure of form *C* of (*R*)-baclofen. In contrast, the cohesion of the apparently less stable form *A* is ensured by π–π inter­actions.

Hydrogen-bond geometry data as well as non-classical C—H⋯Cl inter­action data are summarized in Table 1 ▸.

## Database survey

Using the CSD database (version 5.42 updates 2 and 3; Groom *et al.*, 2016 ▸), a search for the title compound’s structure and names used in this article was conducted with *CONQUEST* (version 2021.2.0; Bruno *et al.*, 2002 ▸).

While the crystal structures of (*R*)- and (*S*)-baclofenium hydro­chloride were reported in the early 1980s (Chang *et al.*, 1981 ▸, 1982 ▸; refcodes: CRBMZB, CRBMZC10), studies on the phase behavior of pure baclofen have gained attention just recently. This is particularly relevant for the crystal structure of enanti­omerically pure (*R*)-baclofen since X-ray powder diffraction studies were recently described by Couvrat *et al.* (2021 ▸). A total of three polymorphic forms (*A*, *B* and *C*) of (*R*)-baclofen were analyzed by X-ray powder diffraction, form *C* being identified as previously unknown. Based on this nomenclature, the crystal structure of form *C* is reported in this study. For the crystal structure of racemic baclofen, see Maniukiewicz *et al.* (2016 ▸; refcode: AQEKUE). A further array of racemic baclofenium co-crystal structures with various carb­oxy­lic acids were published by Báthori & Kilinkissa (2015 ▸; refcodes: LUSXAA, LUSXEE, LUSXII, LUSXUU, LUSXOO, LUSYAB) and Malapile *et al.* (2021 ▸; refcodes: LABJIL, LABJOR, LABJUX, LABKAE, LABKEI, LABKIM, LABKOS). Additionally, Gendron *et al.* (2019 ▸; refcode: WONSIE01) presented the crystal structure of (*R*)-baclofen hydrogenium maleate.

## Synthesis and crystallization

Crystals of the title compound were grown from a saturated aqueous solution containing enanti­opure (*R*)-baclofen, which was evaporated slowly by a stream of dry argon at 313 K. The purity of the (*R*)-baclofen was verified *via* ^1^H NMR. Enanti­opure (*R*)-baclofen was purchased from *abcr GmbH* (Karlsruhe, Germany) under the name (*R*)-4-amino-3-(4-chloro­phen­yl)butanoic acid.

## Refinement

Crystal data, data collection and structure refinement details are summarized in Table 2 ▸. The N-bound hydrogen atoms were found in difference syntheses, and refined freely. All C-bound H atoms were positioned geometrically and refined using a riding model, with C—H = 0.99 Å (methyl­ene groups), 1.00 Å (methine groups) or 0.95 Å (aryl CH) and with *U*_iso_(H) = 1.2*U*_eq_(C) (methyl­ene groups, aryl CH, methine groups). The structure was refined as a two-component inversion twin (BASF 0.04470).

## Supplementary Material

Crystal structure: contains datablock(s) I. DOI: 10.1107/S2056989021012809/yz2013sup1.cif

Structure factors: contains datablock(s) I. DOI: 10.1107/S2056989021012809/yz2013Isup2.hkl

Supporting information file. DOI: 10.1107/S2056989021012809/yz2013Isup3.cml

CCDC reference: 2125567

Additional supporting information:  crystallographic information; 3D view; checkCIF report

## Figures and Tables

**Table 1:** Hydrogen-bond geometry (Å, °)

*D*—H⋯*A*	*D*—H	H⋯*A*	*D*⋯*A*	*D*—H⋯*A*
N1—H1*A*⋯O2^i^	0.91 (3)	1.91 (3)	2.820 (2)	176 (3)
N1—H1*B*⋯O2^ii^	0.93 (3)	1.80 (3)	2.7149 (19)	168 (2)
N1—H1*C*⋯O1^iii^	0.85 (3)	1.93 (3)	2.775 (2)	174 (3)
N1—H1*B*⋯Cl1^iv^	0.93 (3)	2.95 (2)	3.3192 (14)	105.3 (16)
C4—H4*A*⋯Cl1^v^	0.99	2.73	3.6306 (19)	152
C4—H4*B*⋯Cl1^vi^	0.99	2.81	3.5668 (19)	134

Symmetry codes: (i) x+{\script{1\over 2}}, -y+{\script{1\over 2}}, -z+1; (ii) x, y+1, z; (iii) x-{\script{1\over 2}}, -y+{\script{1\over 2}}, -z+1; (iv) -x+{\script{3\over 2}}, -y+1, z-{\script{1\over 2}}; (v) -x+1, y+{\script{1\over 2}}, -z+{\script{3\over 2}}; (vi) -x+2, y+{\script{1\over 2}}, -z+{\script{3\over 2}}.

**Table 2:** Experimental details

Crystal data	
Chemical formula	C_10_H_12_ClNO_2_
*M*_r_	213.66
Crystal system, space group	Orthorhombic, *P*2_1_2_1_2_1_
Temperature (K)	123
*a*, *b*, *c* (Å)	6.8913 (5), 7.6898 (5), 19.7527 (14)
*V* (Å^3^)	1046.75 (13)
*Z*	4
Radiation type	Mo *K*α
μ (mm^−1^)	0.34
Crystal size (mm)	0.27 × 0.19 × 0.16

Data collection	
Diffractometer	Bruker D8 QUEST diffractometer
Absorption correction	Multi-scan (*SADABS2016/2*; Krause *et al.*, 2015 ▸)
*T*_min_, *T*_max_	0.662, 0.747
No. of measured, independent and observed [*I* > 2σ(*I*)] reflections	23624, 3796, 3447
*R*_int_	0.042
(sin θ/λ)_max_ (Å^−1^)	0.756

Refinement	
*R*[*F* ^2^ > 2σ(*F* ^2^)], *wR*(*F* ^2^), *S*	0.033, 0.082, 1.07
No. of reflections	3796
No. of parameters	140
H-atom treatment	H atoms treated by a mixture of independent and constrained refinement
Δρ_max_, Δρ_min_ (e Å^−3^)	0.36, −0.30
Absolute structure	Refined as an inversion twin
Absolute structure parameter	0.04 (6)

Computer programs: *APEX2* and *APEX2* (Bruker, 2003 ▸), *SAINT* (Bruker, 2003 ▸), *SHELXT* (Sheldrick, 2015*a* ▸), *SHELXL2014/7* (Sheldrick, 2015*b* ▸) and *ORTEP-3 for Windows* (Farrugia, 2012 ▸).

## supplementary crystallographic information

### Crystal data

**Table:**

C_10_H_12_ClNO_2_	*D*_x_ = 1.356 Mg m^−^^3^
*M**_r_* = 213.66	Mo *K*α radiation, λ = 0.71073 Å
Orthorhombic, *P*2_1_2_1_2_1_	Cell parameters from 9871 reflections
*a* = 6.8913 (5) Å	θ = 2.8–33.0°
*b* = 7.6898 (5) Å	µ = 0.34 mm^−^^1^
*c* = 19.7527 (14) Å	*T* = 123 K
*V* = 1046.75 (13) Å^3^	Block, colourless
*Z* = 4	0.27 × 0.19 × 0.16 mm
*F*(000) = 448	

### Data collection

**Table:**

Bruker D8 QUEST diffractometer	3796 independent reflections
Radiation source: microfocus sealed tube	3447 reflections with *I* > 2σ(*I*)
Detector resolution: 10.4167 pixels mm^-1^	*R*_int_ = 0.042
phi and ω scans	θ_max_ = 32.5°, θ_min_ = 2.1°
Absorption correction: multi-scan (*SADABS2016/2*; Krause *et al.*, 2015)	*h* = −10→10
*T*_min_ = 0.662, *T*_max_ = 0.747	*k* = −11→11
23624 measured reflections	*l* = −29→29

### Refinement

**Table:**

Refinement on *F*^2^	Hydrogen site location: mixed
Least-squares matrix: full	H atoms treated by a mixture of independent and constrained refinement
*R*[*F*^2^ > 2σ(*F*^2^)] = 0.033	*w* = 1/[σ^2^(*F*_o_^2^) + (0.0372*P*)^2^ + 0.2629*P*] where *P* = (*F*_o_^2^ + 2*F*_c_^2^)/3
*wR*(*F*^2^) = 0.082	(Δ/σ)_max_ = 0.001
*S* = 1.07	Δρ_max_ = 0.36 e Å^−^^3^
3796 reflections	Δρ_min_ = −0.30 e Å^−^^3^
140 parameters	Absolute structure: Refined as an inversion twin
0 restraints	Absolute structure parameter: 0.04 (6)
Primary atom site location: dual	

### Special details

**Table:**

Geometry. All esds (except the esd in the dihedral angle between two l.s. planes) are estimated using the full covariance matrix. The cell esds are taken into account individually in the estimation of esds in distances, angles and torsion angles; correlations between esds in cell parameters are only used when they are defined by crystal symmetry. An approximate (isotropic) treatment of cell esds is used for estimating esds involving l.s. planes. Least-squares planes (x,y,z in crystal coordinates) and deviations from them (* indicates atom used to define plane) 3.7985 (0.0046) x + 6.4064 (0.0034) y + 0.9085 (0.0146) z = 5.2962 (0.0108) * 0.0030 (0.0012) C5 * -0.0067 (0.0012) C6 * 0.0039 (0.0013) C7 * 0.0025 (0.0013) C8 * -0.0062 (0.0014) C9 * 0.0035 (0.0014) C10 2.7365 (0.0020) H9_$8 3.2353 (0.0036) H6_$9 Rms deviation of fitted atoms = 0.0046 3.7985 (0.0045) x - 6.4064 (0.0034) y + 0.9085 (0.0145) z = 0.4604 (0.0136) Angle to previous plane (with approximate esd) = 67.162 ( 0.045 ) * -0.0030 (0.0012) C5_$8 * 0.0067 (0.0012) C6_$8 * -0.0039 (0.0013) C7_$8 * -0.0025 (0.0014) C8_$8 * 0.0062 (0.0014) C9_$8 * -0.0035 (0.0014) C10_$8 Rms deviation of fitted atoms = 0.0046 3.7985 (0.0045) x + 6.4064 (0.0034) y - 0.9085 (0.0145) z = 7.2622 (0.0039) Angle to previous plane (with approximate esd) = 66.899 ( 0.045 ) * -0.0030 (0.0012) C5_$6 * 0.0067 (0.0012) C6_$6 * -0.0039 (0.0013) C7_$6 * -0.0025 (0.0013) C8_$6 * 0.0062 (0.0014) C9_$6 * -0.0035 (0.0014) C10_$6 Rms deviation of fitted atoms = 0.0046
Refinement. Refined as a 2-component inversion twin (BASF 0.04470).

### Fractional atomic coordinates and isotropic or equivalent isotropic displacement parameters (Å^2^)

**Table:**

	*x*	*y*	*z*	*U*_iso_*/*U*_eq_	
Cl1	0.90826 (7)	0.16066 (7)	0.88304 (2)	0.02370 (11)	
N1	0.5842 (2)	0.60260 (18)	0.52352 (7)	0.0134 (2)	
O1	0.76064 (19)	0.02092 (17)	0.54492 (7)	0.0194 (3)	
O2	0.45342 (19)	−0.06602 (16)	0.53636 (7)	0.0178 (3)	
C1	0.5825 (3)	0.0466 (2)	0.54937 (7)	0.0123 (3)	
C2	0.5085 (2)	0.2255 (2)	0.57066 (9)	0.0147 (3)	
H2A	0.4081	0.2098	0.6060	0.018*	
H2B	0.4454	0.2809	0.5311	0.018*	
C3	0.6643 (2)	0.3497 (2)	0.59801 (8)	0.0124 (3)	
H3	0.7818	0.3378	0.5687	0.015*	
C4	0.5966 (3)	0.5388 (2)	0.59429 (8)	0.0139 (3)	
H4A	0.4674	0.5490	0.6159	0.017*	
H4B	0.6882	0.6130	0.6199	0.017*	
C5	0.7231 (2)	0.3034 (2)	0.67012 (8)	0.0134 (3)	
C6	0.6148 (2)	0.3582 (2)	0.72594 (8)	0.0160 (3)	
H6	0.5005	0.4248	0.7190	0.019*	
C7	0.6719 (3)	0.3166 (2)	0.79192 (8)	0.0173 (3)	
H7	0.5986	0.3560	0.8297	0.021*	
C8	0.8370 (3)	0.2172 (2)	0.80119 (8)	0.0176 (3)	
C9	0.9459 (3)	0.1590 (3)	0.74684 (9)	0.0223 (4)	
H9	1.0580	0.0896	0.7540	0.027*	
C10	0.8886 (3)	0.2037 (2)	0.68165 (8)	0.0200 (3)	
H10	0.9639	0.1655	0.6442	0.024*	
H1A	0.701 (5)	0.586 (4)	0.5031 (16)	0.044 (8)*	
H1B	0.555 (4)	0.721 (3)	0.5245 (11)	0.022 (6)*	
H1C	0.490 (4)	0.557 (4)	0.5021 (14)	0.029 (7)*	

### Atomic displacement parameters (Å^2^)

**Table:**

	*U*^11^	*U*^22^	*U*^33^	*U*^12^	*U*^13^	*U*^23^
Cl1	0.02181 (19)	0.0335 (2)	0.01578 (16)	0.0041 (2)	−0.00328 (16)	0.00057 (16)
N1	0.0134 (6)	0.0094 (6)	0.0174 (6)	0.0003 (5)	−0.0012 (5)	0.0000 (4)
O1	0.0152 (6)	0.0173 (6)	0.0258 (6)	0.0016 (5)	0.0031 (5)	−0.0055 (5)
O2	0.0167 (6)	0.0096 (5)	0.0272 (6)	−0.0011 (4)	−0.0027 (5)	−0.0011 (5)
C1	0.0156 (7)	0.0097 (6)	0.0117 (6)	0.0006 (6)	0.0003 (6)	0.0009 (5)
C2	0.0147 (7)	0.0091 (6)	0.0202 (7)	0.0006 (6)	−0.0025 (6)	−0.0018 (6)
C3	0.0130 (6)	0.0099 (6)	0.0144 (6)	0.0013 (6)	−0.0013 (5)	−0.0010 (5)
C4	0.0159 (7)	0.0104 (6)	0.0155 (6)	0.0015 (6)	−0.0012 (6)	−0.0012 (5)
C5	0.0133 (7)	0.0100 (7)	0.0169 (7)	0.0006 (5)	−0.0013 (5)	−0.0010 (5)
C6	0.0146 (7)	0.0146 (7)	0.0187 (7)	0.0028 (6)	0.0015 (5)	0.0012 (6)
C7	0.0186 (7)	0.0171 (8)	0.0163 (7)	0.0010 (7)	0.0029 (6)	0.0004 (6)
C8	0.0188 (8)	0.0188 (8)	0.0151 (7)	0.0001 (6)	−0.0025 (6)	0.0002 (6)
C9	0.0200 (9)	0.0276 (9)	0.0194 (7)	0.0115 (8)	−0.0034 (6)	0.0002 (7)
C10	0.0188 (8)	0.0243 (9)	0.0169 (7)	0.0090 (7)	−0.0015 (6)	−0.0030 (6)

### Geometric parameters (Å, º)

**Table:**

Cl1—C8	1.7445 (17)	C3—H3	1.0000
N1—C4	1.484 (2)	C4—H4A	0.9900
N1—H1A	0.91 (3)	C4—H4B	0.9900
N1—H1B	0.93 (3)	C5—C10	1.394 (2)
N1—H1C	0.85 (3)	C5—C6	1.396 (2)
O1—C1	1.247 (2)	C6—C7	1.398 (2)
O2—C1	1.268 (2)	C6—H6	0.9500
C1—C2	1.526 (2)	C7—C8	1.383 (3)
C2—C3	1.535 (2)	C7—H7	0.9500
C2—H2A	0.9900	C8—C9	1.384 (2)
C2—H2B	0.9900	C9—C10	1.390 (2)
C3—C5	1.523 (2)	C9—H9	0.9500
C3—C4	1.529 (2)	C10—H10	0.9500
			
C4—N1—H1A	109 (2)	C3—C4—H4A	109.2
C4—N1—H1B	108.4 (14)	N1—C4—H4B	109.2
H1A—N1—H1B	110 (3)	C3—C4—H4B	109.2
C4—N1—H1C	112.1 (19)	H4A—C4—H4B	107.9
H1A—N1—H1C	114 (2)	C10—C5—C6	118.31 (15)
H1B—N1—H1C	104 (2)	C10—C5—C3	119.94 (14)
O1—C1—O2	124.61 (16)	C6—C5—C3	121.76 (14)
O1—C1—C2	119.43 (15)	C5—C6—C7	121.13 (15)
O2—C1—C2	115.95 (15)	C5—C6—H6	119.4
C1—C2—C3	115.09 (14)	C7—C6—H6	119.4
C1—C2—H2A	108.5	C8—C7—C6	118.76 (15)
C3—C2—H2A	108.5	C8—C7—H7	120.6
C1—C2—H2B	108.5	C6—C7—H7	120.6
C3—C2—H2B	108.5	C7—C8—C9	121.46 (16)
H2A—C2—H2B	107.5	C7—C8—Cl1	119.47 (13)
C5—C3—C4	110.37 (13)	C9—C8—Cl1	119.07 (14)
C5—C3—C2	111.71 (14)	C8—C9—C10	119.01 (16)
C4—C3—C2	111.18 (13)	C8—C9—H9	120.5
C5—C3—H3	107.8	C10—C9—H9	120.5
C4—C3—H3	107.8	C9—C10—C5	121.32 (16)
C2—C3—H3	107.8	C9—C10—H10	119.3
N1—C4—C3	112.15 (12)	C5—C10—H10	119.3
N1—C4—H4A	109.2		
			
O1—C1—C2—C3	−11.2 (2)	C10—C5—C6—C7	0.9 (3)
O2—C1—C2—C3	169.78 (14)	C3—C5—C6—C7	−179.16 (16)
C1—C2—C3—C5	−76.36 (17)	C5—C6—C7—C8	−1.0 (3)
C1—C2—C3—C4	159.86 (13)	C6—C7—C8—C9	0.1 (3)
C5—C3—C4—N1	165.95 (14)	C6—C7—C8—Cl1	−178.86 (14)
C2—C3—C4—N1	−69.51 (18)	C7—C8—C9—C10	0.8 (3)
C4—C3—C5—C10	−138.39 (16)	Cl1—C8—C9—C10	179.81 (16)
C2—C3—C5—C10	97.37 (19)	C8—C9—C10—C5	−0.9 (3)
C4—C3—C5—C6	41.7 (2)	C6—C5—C10—C9	0.0 (3)
C2—C3—C5—C6	−82.53 (19)	C3—C5—C10—C9	−179.86 (18)

### Hydrogen-bond geometry (Å, º)

**Table:**

*D*—H···*A*	*D*—H	H···*A*	*D*···*A*	*D*—H···*A*
N1—H1*A*···O2^i^	0.91 (3)	1.91 (3)	2.820 (2)	176 (3)
N1—H1*B*···O2^ii^	0.93 (3)	1.80 (3)	2.7149 (19)	168 (2)
N1—H1*C*···O1^iii^	0.85 (3)	1.93 (3)	2.775 (2)	174 (3)
N1—H1*B*···Cl1^iv^	0.93 (3)	2.95 (2)	3.3192 (14)	105.3 (16)
C4—H4*A*···Cl1^v^	0.99	2.73	3.6306 (19)	152
C4—H4*B*···Cl1^vi^	0.99	2.81	3.5668 (19)	134

Symmetry codes: (i) *x*+1/2, −*y*+1/2, −*z*+1; (ii) *x*, *y*+1, *z*; (iii) *x*−1/2, −*y*+1/2, −*z*+1; (iv) −*x*+3/2, −*y*+1, *z*−1/2; (v) −*x*+1, *y*+1/2, −*z*+3/2; (vi) −*x*+2, *y*+1/2, −*z*+3/2.
